# A Sensor Fault-Tolerant Accident Diagnosis System

**DOI:** 10.3390/s20205839

**Published:** 2020-10-15

**Authors:** Jeonghun Choi, Seung Jun Lee

**Affiliations:** Ulsan National Institute of Science and Technology, 50 UNIST-gil, Ulju-gun, Ulsan 44919, Korea; jhchoi@unist.ac.kr

**Keywords:** sensor fault mitigation, sensor fault-tolerant accident diagnosis, recurrent neural networks, signal reconstruction

## Abstract

Emergency situations in nuclear power plants are accompanied by an automatic reactor shutdown, which gives a big task burden to the plant operators under highly stressful conditions. Diagnosis of the occurred accident is an essential sequence for optimum mitigations; however, it is also a critical source of error because the results of accident identification determine the task flow connected to all subsequent tasks. To support accident identification in nuclear power plants, recurrent neural network (RNN)-based approaches have recently shown outstanding performances. Despite the achievements though, the robustness of RNN models is not promising because wrong inputs have been shown to degrade the performance of RNNs to a greater extent than other methods in some applications. In this research, an accident diagnosis system that is tolerant to sensor faults is developed based on an existing RNN model and tested with anticipated sensor errors. To find the optimum strategy to mitigate sensor error, Missforest, selected from among various imputation methods, and gated recurrent unit with decay (GRUD), developed for multivariate time series imputation based on the RNN model, are compared to examine the extent that they recover the diagnosis accuracies within a given threshold.

## 1. Introduction

In safety-critical systems, a prompt reaction to anomalies is a crucial factor to minimize any related consequences. The nuclear fuel generating fission energy in nuclear power plants (NPPs) is a possible threat to the public in case of a large release of radioactive materials following a nuclear accident. In terms of component failures and external threats, various systems need to be prepared for maintaining plant safety and stability. Against expected accidents, NPPs have several safety systems that are initiated by process parameters exceeding threshold values or manual operations by plant operators. Responses to emergency situations follow specific procedures containing sequential tasks. Depending on the accident symptoms, accident diagnosis is conducted to specify the exact type of occurred accident in order to know how to mitigate the event. Diagnosis is therefore crucial because it determines the particular optimal recovery procedures (ORPs) that contain the essential mitigation tasks [1,2]. Diagnosis procedures give intuitive logics for identifying accidents based on a series of symptom checks, but can be a demanding task for the plant operators because the early phases of an emergency situation may affect the accident consequences. In this regard, a wrong diagnosis would lead to selecting the wrong ORPs, which could result in multiple human errors.

Plant states are monitored via myriad sensors connected to components, with the sensor values acting as the basic elements for the state awareness of plant operators and also the cause of automatic safety system actuation. In this regard, faulty information from sensors may confuse the operators and even lead to misguided judgments. Indeed, misdiagnosis from sensor faults has been discussed as a type of deterioration factor in severe accidents such as the Three Mile Island (TMI-2) and Fukushima accidents [3,4]. The TMI-2 accident in particular represents a case in which sensor faults induced a critical misdiagnosis error, as follows. Electromagnetic relief valves, also called pilot-operated relief valves (PORVs), were accidentally stuck open; however, the related indicator showed that the valves were in a closed state. From this error, the operator incorrectly recognized the situation and turned off the safety-injection system which had been automatically actuated to cool down the reactor core [5].

To reduce the number of required tasks and still achieve accurate accident diagnosis, several methods have been suggested mainly based on data-driven models [6,7,8]. These methods require longitudinal multivariate plant parameters containing the accident symptoms. However, countermeasures for sensor faults during the accident sequence are not discussed in either existing diagnosis procedures or developed diagnosis models. In this context, with a lack of consideration about diagnosis failure from sensor faults, current diagnosis models seem vulnerable to sensor errors.

Related research concerning sensor faults in the nuclear field is represented by online monitoring techniques using auto-associative kernel regressions, fuzzy similarity, singular value decomposition, and neural networks [9,10,11,12]. The target of most of these approaches is normal operation though, meaning that transient situations including accidents have yet to be studied. To consider sensor health monitoring during emergency situations, a sensor fault detection system was recently suggested using long short-term memory networks [13]. This work showed that sensor states during a typical accident sequence can be monitored by a single machine learning model, and thus that sensor fault information can be provided in emergency situations.

To enhance the applicability of accident diagnosis models, measures to mitigate sensor faults are essential. In the present work, a sensor fault-tolerant accident diagnosis system is developed to support the diagnosis of nuclear accidents containing sensor faults. The system comprises sensor fault detection and mitigation subsystems. Starting from a reactor trip, which occurs autonomously in an emergency situation, the directly adapted faulty sensor detection subsystem developed in a previous work monitors the sensor state, and upon detection of sensor error, the sensor fault mitigation subsystem initiates. In developing the mitigation subsystem, various mitigation strategies were compared, including data replacement for the faulty sensor as well as weight decay of the inner cell of the recurrent neural network. Related insights are discussed based on the required computation times and the resulting diagnosis accuracy of the tested strategies.

## 2. Accident Diagnosis in an Emergency Situation

### 2.1. Early Responses to Reactor Trip

In NPPs, several operating procedures are prepared for diverse scales of anomalies. As a response to individual component malfunctions or potential threats to the reactor core integrity, alarm response procedures or abnormal operating procedures are performed. Such kinds of frequent incidents are called anticipated operational occurrences. More serious deviations beyond the anticipated operational occurrences are defined as “accidents”. In accident conditions, the reactor automatically trips when the reactor protection system detects deviations from predefined setpoints. In a design basis accident, the situation has no radiological impacts at all or no impact outside the exclusion area, since the incident can be fully mitigated by the equipped safety features. Certain accident situations are called emergency situations, in which emergency operating procedures (EOPs) must be performed. The EOP provides procedural guidance with the focus to prevent core damage. According to the accident type, which varies by the location of a break or the loss of particular feedwater sources, optimum responses are completely different; thus, the diagnosis of the accident is essential for arranging the proper mitigation tasks [1].

The diagnosis task includes a range of miscellaneous work by the plant operators. Julius et al. classified errors of commission into three types [14]: global misdiagnosis, local misdiagnosis, and slip. Global misdiagnosis refers to an error of commission resulting from the selection of inappropriate response procedures. Local misdiagnosis refers to an error of commission resulting from an intentional inappropriate measure, which may be caused by an incorrect understanding of the related procedure and information. A slip is an error caused by unintentional and inappropriate performance when performing work. According to these classifications, misdiagnosis by plant operators includes not only the wrong selection of the ORPs but also small-scale misdiagnoses such as an incorrect understanding of procedural tasks or related information. While both types are a possible candidate for human errors, global misdiagnosis could result in more serious consequences because it contains numerous tasks for mitigating the accident.

**Figure 1:**
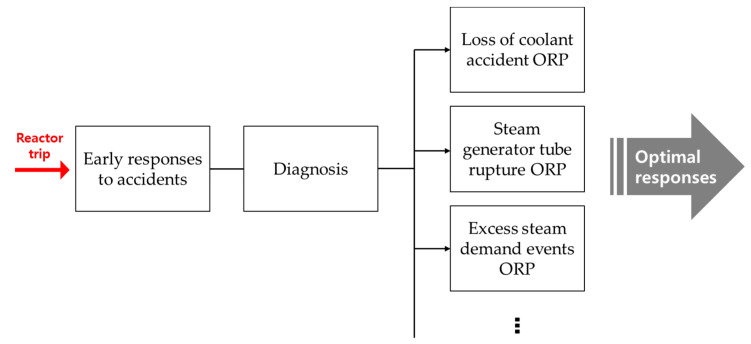
Emergency operating procedure (EOP) package (symptom-based).

Emergency situations are unfamiliar to plant operators since they rarely occur in practice; thus, performing an EOP is a complex and stressful task. Operators need to specify the type of accident to know the optimum response while surrounded by numerous alarms. To make a proper accident diagnosis, proper situational awareness is essential to check the various accident symptoms. To guide the operator in a standardized way, the diagnosis procedure provides conditional logics to specify the accident type from its symptoms. Figure 1 shows the diagnosis flow in the EOP package, which includes the early responses to an accident, the diagnosis procedure, and the appropriate ORPs depending on the particular accident [15]. This structure is vulnerable to wrong process parameters from sensor errors. A single faulty transition within a procedure could result in a failure of diagnosis, thereby preventing the optimum response to occur as well as the commission of unnecessary tasks or even harmful actions. Ensuring robust and accurate accident diagnosis performance in NPPs is indispensable for safe operations.

### 2.2. Accident Diagnosis Methods for Nuclear Power Plants (NPPs)

Indicating the accident type is a crucial step to mitigate an emergency. The emergency situation gives the operator a lot of pressure and stress and invites potential human errors due to the complicated diagnosis procedures [16]. Accident identification algorithms based on several machine learning or statistical model-based methods have been suggested to achieve stable and quick diagnosis outputs. Diagnosis tasks include the checking of multiple process parameters in terms of trends or values exceeding thresholds, or in other words, knowledge of multivariate parameters is needed. Accident diagnosis algorithms thus need to classify multivariate data with temporal analysis. For this, in the early stages of development, artificial neural networks (ANNs) [17], neuro-fuzzy networks [18,19,20], and knowledge-based expert systems [21] were suggested. More recently, the rapid growth of neural networks has improved the diagnostic performance of the algorithms, with fine performances seen from deep neural networks [22], recurrent neural networks (RNNs) [23], and convolutional neural networks (CNNs) [24]. Additionally, hidden Markov models, pattern recognition, and Bayesian belief networks have also been used to identify accident types [25,26,27].

## 3. Fault-Tolerant Accident Diagnosis System Framework

**Figure 2:**
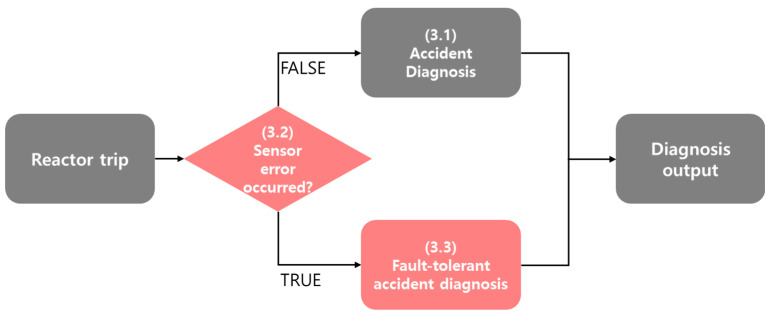
Overall framework for the proposed sensor fault-tolerant accident diagnosis system.

Our system consists of two subsystems: a sensor fault detection system and a sensor fault-tolerant mitigation system, as shown in Figure 2. Sensor fault monitoring is performed at the frontline of the system, initiated upon a reactor trip (3.2 in Figure 2). In the absence of sensor faults, an accident diagnosis system generates output to identify the accident (3.1 in Figure 2). If the sensor fault monitoring system detects sensor error, the faulty information is transferred to the sensor fault mitigation system (3.3 in Figure 2), where via data imputation methods, the faulty sensor data are substituted for or their influence is weakened. Finally, fault-tolerant accident diagnosis results are generated.

### 3.1. Accident Diagnosis Algorithm Using Gated Recurrent Unit (GRU)

In the nuclear field, among the developed accident diagnosis algorithms, deep neural network-based techniques have successfully generated proper accident labels from simulated data [22,28]. Recently, accident diagnosis models for emergency situations using RNN-based algorithms have been suggested [23]. These types of RNNs use more advanced models, such as long short-term memory networks or the gated recurrent unit (GRU), to classify accident or abnormal data [29,30,31]. 

**Figure 3:**
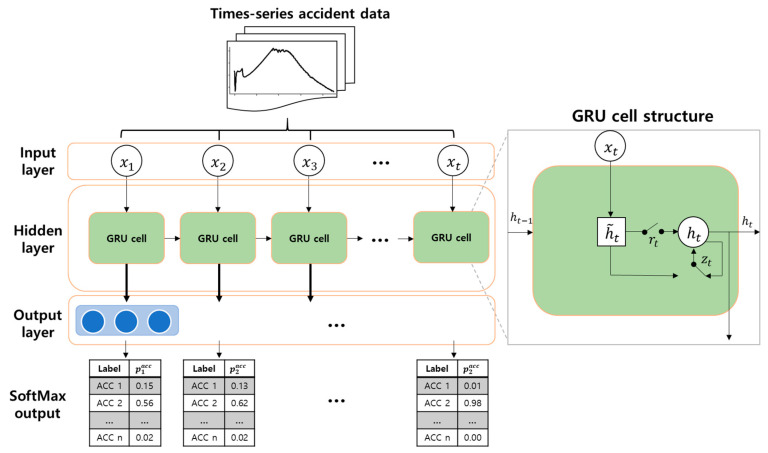
Gated recurrent unit (GRU)-based accident diagnosis algorithm with multivariate time-series input and SoftMax output.

GRU consists of two main gate functions, a reset gate ($r_{t}$) and an update gate ($z_{t}).\;$ The reset gate determines to what extent the previous state ($h_{t-1})$ should be reflected using a sigmoid function, and the update gate decides how to update the present state from the previous state and the input data. The candidate function (${\tilde{h}}_{t}$) and the previous state are modulated by the update gate to determine the present hidden state ($h_{t}$). The determined hidden state is then transferred to the next GRU cell or exits as an output. The formulas for the gate functions, hidden state, and candidate functions are as below:

(1) $$z_{t}=\sigma \left(W_{z}x_{t}+U_{z}h_{t-1}\right)$$

(2) $${\tilde{h}}_{t}=\tanh\left(Wx_{t}+U\left(r_{t}⨀h_{t-1}\right)\right)$$

(3) $$h_{t}=\left(1-z_{t}\right)⨀h_{t-1}+z_{t}⨀{\tilde{h}}_{t}$$

(4) $$r_{t}=\sigma \left(W_{r}x_{t}+U_{r}h_{t-1}\right)$$

where $z_{t},\;{\tilde{h}}_{t},\;r_{t}$ are the update, candidate, and reset gates, respectively, W, U are weighted vectors, and x, h is the input and hidden state, respectively. ⨀ is an element-wise multiplication [32]. Figure 3 depicts the GRU-based accident diagnosis algorithm.

For our system, a GRU-based accident diagnosis algorithm was constructed to check diagnosis performance by testing for performance degradation from faulty inputs. The algorithm has one hidden layer with 64 nodes and applies the Adam optimizer for training [33]. The hyper parameters, including the number of hidden layers and nodes, were determined from a pilot study to achieve sufficient diagnosis performance. The output results are generated with the SoftMax function that generates normalized output with a probability distribution [34]. The formula for SoftMax normalization is as follows:

(5) $$S{\left(z\right)}_{i}=\frac{e^{z_{i}}}{\sum_{j=1}^{K}e^{z_{j}}}$$

These features of GRU are quite suitable for accident diagnosis because of the connections between cells and the forward propagation that transfers contextual information. However, robustness issues in neural network models have been raised, with measures to increase their robustness receiving active attention [35,36,37]. Baraldi et al. (2015) showed higher performance but much lower robustness of an RNN compared to other data-driven methods in energy production data reconstruction [38]. Likewise, Kim et al. (2018) compared the performance of neural network models and found the RNN to have lower robustness with missing values in some cases [39]. From the references, the robustness of the RNN model is not assured, and thus strengthened measures for the threat of possible sensor faults should be prepared to prevent model failure from wrong inputs.

### 3.2. Sensor Fault Detection System [13]

**Figure 4:**
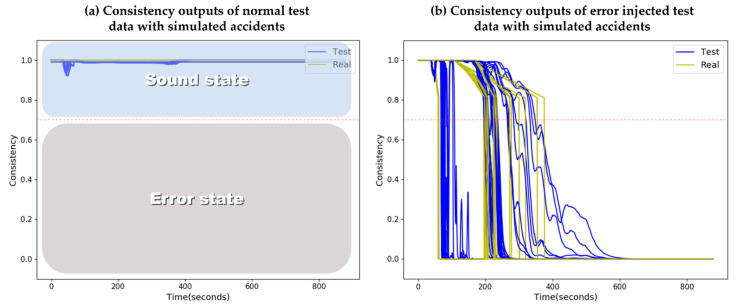
Examples of sensor state identification with (**a**) normal data test results and (**b**) error injected data test results. This figure is adapted from [13].

To account for sensor faults in an emergency situation, Choi et al. (2020) suggested a sensor fault detection system utilizing the notable performance of RNNs with several time-series multivariate data, as a prior study of the present work [13]. The output of the detection system is generated in the form of a consistency index, which is a numerically normalized index of sensor health. The consistency index is maintained around 1 in normal states and decreases following the degree of sensor signal deviations, as shown in Figure 4. From empirical test results in a previous study, the consistency index criteria, as a fault threshold, was determined to have a value of 0.7 considering both the detection speed and uncertainties. By the consistency index, sensor fault information can be derived with masking inputs. Having the same data structure as time-series inputs, masking inputs indicate the absence of data with binary remarks; here, ‘1′ indicates that the sensor value is normally observed, while ‘0′ means that the data is missing. The masking inputs are determined by Equation (6):

(6) $$m_{t}^{i}=\{\begin{array}{c} 1,\;\;\;C_{t}^{i}\geq 0.7\\ 0,\;\;\;C_{t}^{i}<0.7 \end{array}$$

**Figure 5:**
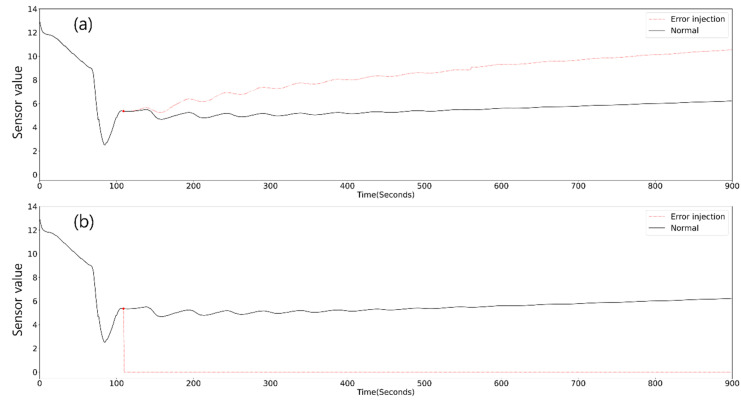
Example trends of (**a**) drift and (**b**) stuck at zero sensor errors.

Sensor error modes were selected in the previous study considering the connections to human error and typicality. Drift and stuck errors were injected to accident data, and their consistency was evaluated. In the present study, the same sensor errors are implemented to check the performance degradation of the diagnosis algorithm. Drift error with 2- and 10-times rates of change with two directions (upward, downward) and the stuck at zero error are injected into the accident data. Figure 5 shows the cases of drift and stuck error that occurred at 100 s. In the pilot study, error injection closer to 0 s generated the largest deviation in the diagnosis results, and thus, all error injection is added at 1 s of the data.

### 3.3. Fault-Tolerant Accident Diagnosis System

From the successful results of the sensor fault detection model in Section 3.1, the sensor states in an accident can be confidently monitored with their information continuously available. In the event that a sensor fault occurs, the incoming data, as inherently unreliable from a faulty sensor, needs to be removed. However, accident diagnosis algorithms based on GRU cannot accept any empty inputs because the GRU structure dictates that each input influences all functions and outputs with their interconnections. To mitigate any missing data, it is therefore essential to estimate the missing values or apply a modified RNN structure. We applied imputation methods, which mean the process of substituting for missing data with estimations, and transformed the GRU model to construct the fault-tolerant accident diagnosis system.

First, simple imputation methods for time-series data were considered including moving window imputation or last observed carried forward imputation. These methods are inappropriate in our case though because a faulty sensor continuously generates deviated data after the fault occurred. In other words, simple approaches cannot reflect the characteristics of multivariate time-series data. To reflect the diverse plant symptoms based on accident type, at least one statistical model needs to be included in the imputation model. Considering general usage and performance, three imputation methods are compared to replace the sensor error data.

#### 3.3.1. K-Nearest Neighbors (KNN)

K-nearest neighbors (KNN) is a popular single imputation method in which only a single calculation is performed. The basic principle of KNN imputation is estimation via an average calculation of multiple neighbors. Based on the *K* parameter setting, the nearest data are grouped with certain distance calculations including Manhattan distance, Euclidian distance, and correlation distance [40]. The missing data are replaced with the weighted average of the nearest neighbors. In our model, the Euclidian distance-based KNN method was used to impute the data.

#### 3.3.2. Multivariate Imputation with Chained Equations (MICE)

To overcome the limitations of single imputation methods, several multiple imputation methods have emerged. The process of multiple imputations is as follows. (1) A simple imputation is conducted (e.g., mean) for every missing data as a place holder; (2) the variable having the largest missing portion is returned to the missing data; (3) the variable is regressed from the other variables; and (4) the regression is repeated until the result converges. Regression models typically include linear, logistic, and Poisson regressions [41]. The particular regression model and convergence criteria differ between MICE software packages; in our model, linear regression with a convergence criteria of Δ <0.1 was applied (i.e., the relative change of the new imputation value from the old imputation value is under 0.1).

#### 3.3.3. Missforest

The Missforest imputation method, originally suggested to handle big data containing missing sections in the medical industry, is a means of multiple imputation that can be applied to both categorical and numerical data [42]. It has a similar imputation process as MICE but with a different regression method; Missforest performs iterative random forest regression, which is a popular machine learning method that constructs numerous decision trees from training and generates a mean regression by ensembling multiple decision trees [43]. The computation time of Missforest can be controlled by the inputs, number of trees, and number of iterations.

In addition to replacing the missing data itself, the structure of the RNN can also be modified to utilize missing data. Structurally, an RNN contains the same parameters in all time-series data, and thus the input data must match the number of data dimensions of the trained model. Since losses of data may accidentally occur for any variable and scale, existing RNN models cannot make an output when inputs are missing. To utilize multivariate time-series data with missing sections, the GRU-decay (GRUD) model has been suggested [44].

#### 3.3.4. GRU-Decay (GRUD)

**Figure 6:**
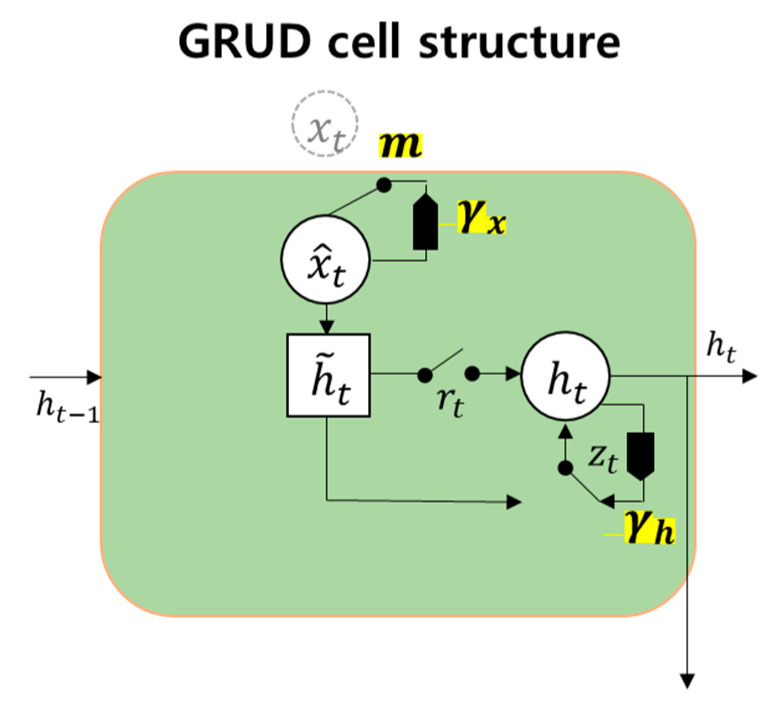
GRU-decay (GRUD) scheme assuming the current input is missing.

The GRUD model adds simple imputation and a weight decay mechanism to the basic GRU structure to reduce the effect of missing data. The decay term (γ) represents the decrease of missing data and is determined from training. The decay mechanism with decay term, γ, is as below:

(7) $$\gamma_{t}=\exp\left(-\max\left(0,W_{\gamma }\sigma_{t}+b_{\gamma }\right)\right)$$

(8) $${\hat{x}}_{t}=m_{t}x_{t}+\left(1-m_{t}\right)\left(\gamma_{x_{t}}x_{t}+\left(1-\gamma_{x_{t}}\right){\overset{˘}{x}}_{t}\right)$$

(9) $${\hat{h}}_{t-1}=\gamma_{h_{t}}⨀h_{t-1}$$

where $\gamma_{t}$ is the decay term and *m* is masking, which shows the missing states of the data. The decay term is determined by an exponentiated negative rectifier with a trained weight and bias of the variable, as in Equation (7). The input and hidden state decrease by the decay term over time as a modified input and hidden state, as in Equations (8) and (9). Figure 6 shows the structure of GRUD with a missing input. Current input $x_{t}$ is not available, and therefore it is substituted by ${\hat{x}}_{t}$, which is decayed from the last observed value to the mean value of the parameter with decay term $\gamma_{x_{t}}$. The hidden state ${\hat{h}}_{t-1}$ decays with decay rate $\gamma_{h_{t}}$, which means a shrinkage of the influence of the input in generating the output. This is advantageous in unified model designs such as combining GRUD with imputation logic in an RNN model that performs actual work, like our system for accident diagnosis classification. In our sensor fault mitigation system, the essential masking input that indicates the missing data [Equation (6)] can come from the fault monitoring system, as described in Section 3.2.

Using the above four approaches, we developed two fault-tolerant diagnosis structures. The next section presents a diagnosis performance comparison of the two structures as mitigation strategies for sensor error in simulated NPP accidents. The first substitutes the missing data with a regression-based imputation method (KNN, MICE, or Missforest) and makes a diagnosis with GRU; Section 4.3 compares the three methods to select the optimum model. The second structure inputs the data with missing sections to GRUD.

## 4. Sensor Fault-Tolerant Diagnosis System Test Results

### 4.1. Data Descriptions

Nuclear accident data were generated from a compact nuclear simulator (CNS) of the Westinghouse 940 MWe pressurized water reactor with a compact scale. Employed CNS was developed by the Korea Atomic Energy Research Institute (KAERI). This simulator has been used as a source of several data-driven machine learning applications in the nuclear field. The CNS can generate emergency or accident data with detailed malfunction options; while it is a simplified one-dimensional model with theoretical assumptions that cannot simulate all accident phenomena, it can generate a large amount of data in a short time [45,46,47,48]. Among the 2217 process parameters the CNS generated, 41 parameters were selected here based on the existent diagnosis procedure in the EOPs and to include parameters that indicate specific accident symptoms. The data acquisition period was set to 900 s by referring to the recommended accident diagnosis time limits in IAEA safety reports [49].

All selected parameters show non-linear and unstable changes in an emergency situation with a reactor trip and the actuation of various safety systems. The data also includes some unexpected phenomena, e.g., oscillation generated from vaporization, with the diverse accident symptoms differing from the detailed malfunction options. Table 1 lists the simulated accidents with the related numbers of datasets.

From five broad categories of possible NPP accidents, data from nine detailed accident sequences were extracted with 1850 data divided by severity or break location that generate various accident features. The loss of coolant accident (LOCA) data was divided into small/medium LOCA and large LOCA by the break size from a reference [50], and the pilot-operated relief valve (PORV) LOCA was added due to its distinctive symptoms from other LOCA types. An excess steam demand event (ESDE), also called a main steam line break, was separated into in- and out-containment since each presents quite different symptoms. As general spurious reactor trip accidents, reactor coolant pump (RCP) failure and reactor protection system (RPS) failure were selected. Among the data, 453 test sets were randomly selected, with the other 1397 used for training and validation.

Before the training of the diagnosis algorithm, min-max normalization [51] was performed on all training and test data based on the collected maximum and minimum variable data from among all datasets for efficient training of the neural network model.

### 4.2. Accident Diagnosis Algorithm Test Results

While the SoftMax function at the end of the GRU model generates numerically normalized output, no exact criteria exist for identifying the states. Even though accidents have diverse symptoms according to their types and scales, many studies have shown that accidents can be successfully identified by RNNs. To evaluate the stability and robustness of diagnosis algorithms, consistently high output of the true accident labels should be generated. For these reasons, we set simple criteria for a thorough evaluation of the diagnosis algorithm. After accident occurrence, sufficient time for the symptoms to present is needed. In previous research, sensor faults were detected within an average of 140 s. Accordingly, the success criterion of accident diagnosis in this work is assumed as when the true SoftMax output maintains the maximum value from 200 s to the end of simulation (900 s).

**Figure 7:**
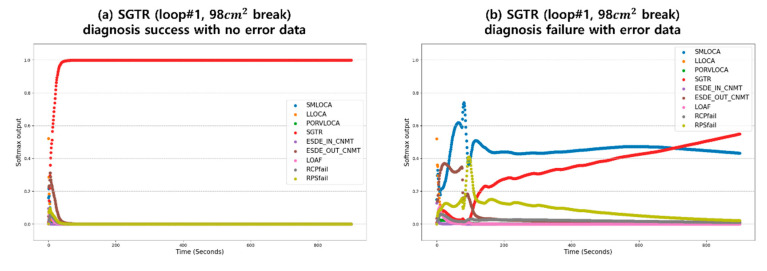
Example trends of (**a**) accident diagnosis success from normal data test, and (**b**) diagnosis failure from error-injected data for the secondary radiation sensor.

Before testing with injected sensor error, the constructed diagnosis algorithm with GRU needs to be initially assessed. The test results of the GRU model are depicted in Table 2 for classifying accident types from among 453 test sets with fault-free data. Unstable trends were observed in S/MLOCA and LLOCA test data, which have break sizes near the boundary value; nevertheless, the output maintained the true diagnosis in all time sequences. Next, the performance degradation of the diagnosis algorithm was analyzed in terms of five selected sensor error modes, giving a total 2265 test data for each sensor. The sensor error data were generated targeting seven process parameters that have diverse trends depending on the accident type and largely influence the diagnosis procedure sequences. Only a single sensor error is assumed in this study. As shown in Table 2, sensor error notably deteriorates the diagnosis performance, with the degree of degradation varying by the particular error-injected sensor. The largest performance drop occurred with the secondary radiation sensor error because this parameter is a crucial factor to discriminate steam generator tube rupture (SGTR) from the other accidents. Figure 7 shows the successful identification of SGTR from normal data and its diagnosis failure from secondary radiation sensor error.

### 4.3. Performance Evaluation of Imputation Models

To select the finest imputation model, accuracies were measured among the imputation methods described in Section 3.3. Mean imputation was also included in the comparative study because it is a base method in all three imputation methods: KNN, MICE, and Missforest. Errors in reconstructed data from the original values were collected in the form of average over time length. Computation times were also collected for the performance comparison because they are a crucial factor in accident situations requiring quick responses. We note that while adjustments of model parameters affect both imputation accuracy and computation time, here such adjustments were not a major determinant of model performance; the parameters for the imputation models (e.g., the number of nearest neighbors for KNN or the number of decision trees for Missforest) were fixed based on pilot tests achieving the best performance in under 20 s of computation time. In this study, as only single sensor faults were assumed, MICE and Missforest deal with single fittings from linear and random forest regression in the tests.

Some process parameters in NPPs contain several zero values, for example, specific radiation alarms that maintain at zero in the absence of a leak of radioactive material. However, the actual values of the sensors need to be inserted as the denominator in error percentage metrics, such as mean absolute percentage error. To handle this problem, the symmetric mean absolute percentage error (sMAPE) metric was suggested by Armstrong [52], which is defined as:

(10) $$sMAPE\;=\frac{1}{n}\overset{n}{\underset{t=1}{\sum }}\frac{2\cdot \left|A_{t}^{v}-F_{t}^{v}\right|}{\left|A_{t}^{v}\right|+\left|F_{t}^{v}\right|}$$

where $A_{t}$ denotes the actual measured value at time *t*, and $F_{t}$ denotes the imputed value. While there are asymmetric issues with sMAPE [53], they are not a concern in actual data with non-negative values. Evaluation with sMAPE was performed here to select the optimum imputation method from among the four models (mean, KNN, MICE, and Missforest). Each method has a crucial parameter to determine the computation time and imputation accuracy. Comparisons of the imputation models were conducted based on the regression of the missing sensor variables from the same sample data as the training dataset; the percentage errors and computation times are listed in Table 3 and Table 4, respectively. The five variables were randomly selected from among the 41 plant parameters (see Section 4.1) to compare the reconstruction performance of the methods independent from the features of the variables.

**Figure 8:**
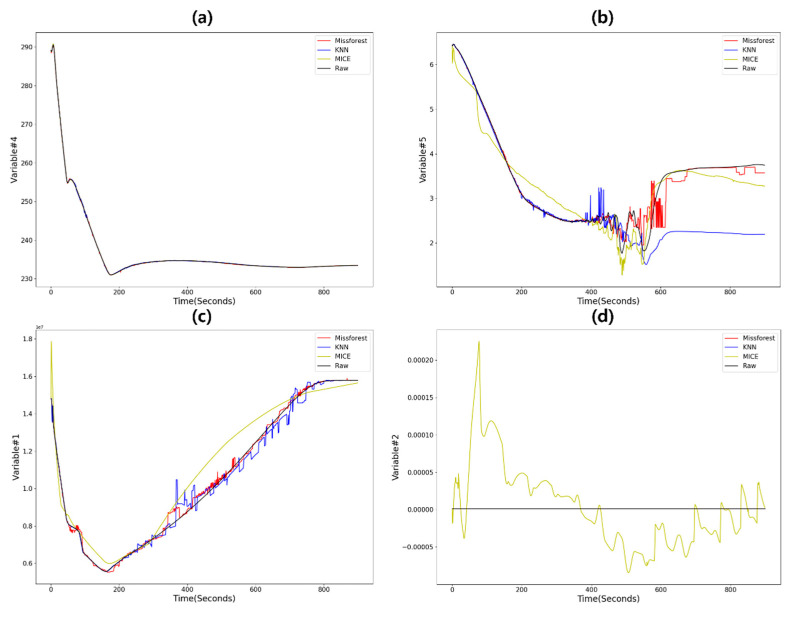
Comparison of imputation results showing (**a**) fine imputation by all models for variable #4 fault, (**b**) the maximum error of K-nearest neighbors (KNN) for variable #5 fault, (**c**) accurate performance of Missforest for variable #1 fault, and (**d**) the maximum error of multivariate imputation with chained equations (MICE) for variable #2 fault.

Overall, Missforest provided the most stable results with the lowest error. MICE showed good performance in a variable with a simple pattern regardless of the accident (Var #4), which seems to be a characteristic of linear regression. But in the case of Var #2 and #3, which contain constant zero data, the performance rapidly decreased, as shown in Figure 8d. While KNN showed fine performance in Min error, its performance was unstable, presenting peaks with unusual tendencies. In terms of the computation time, which as previously stated is a crucial factor in determining applicability to real NPP emergencies, MICE showed an average computation time of under 5 s, Missforest took about 9 s, and KNN about 17 s. Missforest presented various computation times between variables because in this structure, the computation time is determined based on the number of branches; thus, regression of sensor values with diverse trends, such as Var #4, required longer times to calculate.

According to the results, Missforest-based imputation showed the best performance among the tested methods in the aspects of mean error, maximum peak error, and affordable computation time. Thus, for signal reconstruction to replace the missing data from sensor faults, Missforest was selected in this work as the imputation tool.

### 4.4. Fault Mitigation Results

**Figure 9:**
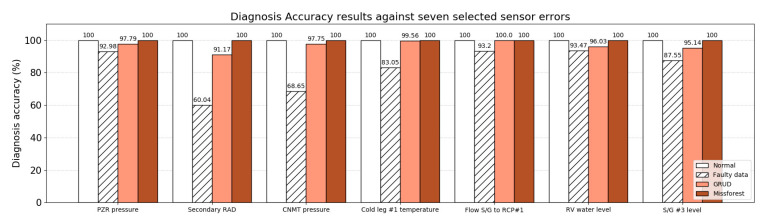
Diagnosis accuracy results of seven sensor errors with error-free data (white), error-injected data (dashed), and the application of GRUD (orange) and Missforest (brown) to the error-injected data.

To check the sensor fault mitigation strategies, both GRUD and Missforest imputation were applied to the unreliable sensor data. Each imputation method was tested with a test set containing seven sensor errors. GRUD showed remarkable performance recovery from error states, as shown in Table 5. The recovered diagnosis accuracy was directly affected by a degraded accuracy; for example, the lowest accuracy among the faulty data for ‘Secondary RAD’ is connected to the lowest recovered accuracy in the mitigated result. In the case of Missforest, all sensor errors were recovered to complete diagnosis accuracy. Performance degradations and recovered diagnosis accuracy concerning the seven sensor errors are arranged in Figure 9.

In summary, we first confirmed that the base diagnosis algorithm with GRU could successfully diagnose the prepared 453 test data with an assumed threshold. After injecting sensor errors, the diagnosis accuracy dropped to an average of 82.71%; i.e., 2742 failure cases occurred out of the total 13,113 test data. As a first mitigation strategy, the GRUD-based fault-tolerant strategy was applied, which achieved a notable accuracy recovery to 96.75%, where 103 failure cases were observed out of the total 3171 test data. As a second strategy, Missforest achieved a complete diagnosis accuracy recovery, in other words 100% with 0 failures out of the 3171 test data. Table 5 lists the final test results.

## 5. Discussion

In emergency situations in medical, aviation, and oil and gas industries, automated systems that support the responses to the situation have been researched applying several data-driven methods [54,55,56,57]. In the nuclear field, where safety is of utmost importance, even though NPPs are equipped with numerous autonomous safety systems, responses to abnormal states still largely depend on the judgments of plant operators. While several NPP accident identification models are being actively researched, faulty input data from a sensor network has yet to be considered. In the present work, it is notable that the GRU-based diagnosis algorithm is not robust to injected sensor faults based on the performance test results, where diagnosis accuracy dropped to about 80% from sensor faults. Both performance degradation and recovery largely depend on sensor features. Because NPPs consist of a large number of components and systems, each accident type may either show specific symptoms or shared symptoms with other accident types, which complicates accident diagnosis. It was found in Section 4.2 that one specific sensor parameter can be crucial to distinguish two different accident types, and thus related sensor errors resulted in a significant deterioration of the accuracy of the accident diagnosis system. Specifically, secondary radiation is a crucial factor for distinguishing SGTR from LOCA, and containment pressure is what divides in/out containment ESDE accidents; in these sensor error tests, performance degradations down to 60.05% and 68.65% accuracies were observed. Error mitigation strategies to cover these occasions are therefore needed to back up diagnosis algorithm applications.

Among the two tested mitigation strategies, Missforest showed a complete recovery with 100% diagnosis accuracy, while GRUD showed 96.75% accuracy, indicating a lack of full recovery from the sensor faults. Despite this, GRUD has advantages in computation time and code complexity. Because a decay mechanism is included in the GRUD structure, computation time is only required for simple imputations. In contrast, Missforest required an average computational time of 9.06 s (SD = 2.552) to generate the imputed data. In an emergency situation requiring prompt responses, this additional calculation time might critically delay the appropriate measures for alleviating the accident. To apply our sensor fault-tolerant diagnosis system in real plants, this trade-off between computational time and performance needs to be carefully examined.

## 6. Conclusions

Machine learning-based data-driven methods are being actively researched for fault identification in the nuclear field. For real application, the developed diagnosis algorithms need to be robust to possible sensor anomalies such as sensor faults and noise. Based on a previously developed sensor fault detection scheme for a nuclear accident, a sensor fault-tolerant accident diagnosis system was constructed in the present work to ensure appropriate diagnostic outputs. The accident diagnosis algorithm was developed based on GRU and validated with CNS data from nine potential NPP accidents. Diagnosis performance degradation from injected sensor errors was observed in the error test data. To select the most appropriate imputation method, the diagnostic performance of three approaches were compared. Results showed the Missforest model and the GRUD model to most successfully recover the degradation from sensor errors.

As an advisory support system, this work is believed to provide plant operators with properly identified information during accident progression. Moreover, the developed fault-tolerant structure can also be applied to NPP abnormal situations and start-up and shutdown operations, as well as other industries requiring process parameter-based reactions sensitive to sensor faults.

To further improve the diagnosis performance of the GRUD-based system, a more developed GRUD structure should be explored. Recently developed multi-directional and bi-directional RNNs allow for the consideration of reverse directional or row-wise contextual situations with decay mechanisms for missing data [58,59]. Future work will apply these types of RNNs to our model in place of the basic GRUD. In addition, our system only assumed error data from a single sensor fault with no operator manipulations in an emergency state. In reality, human operator actions following judgments at random moments will influence the process parameters, while simultaneous sensor errors may also occur. To address these issues, a larger database needs to be constructed for more comprehensive model training and testing before applications of our sensor fault-tolerant accident diagnosis system.

## Figures and Tables

**Table 1:** Number of training and test sets for the nine simulated accidents.

Accident Type	Detailed Accident Type	Accident Label	No. of Training Sets	No. of Test Sets
Loss of coolant accident	Small/medium LOCA	S/MLOCA	228	72
	Large LOCA	LLOCA	390	126
	PORV LOCA	PORVLOCA	54	17
Steam generator tube rupture	Steam generator tube rupture	SGTR	111	36
Excess steam demand event	In-containment ESDE	ESDE_IN_CNMT	216	72
	Out-containment ESDE	ESDE_OUT_CNMT	186	70
Loss of all feedwater	Loss of all feedwater	LOAF	112	38
Reactor trip	Reactor coolant pump failure	RCP fail	50	11
	Reactor protection system failure	RPS fail	50	11
**Total**			**1397**	**453**

**Table 2:** Diagnosis accuracy of GRU with error-injected and no-error sensor data.

Unit: %	PZR Pressure	Secondary RAD	CNMT Pressure	Cold Leg #1 Temp.	Flow S/G to RCP #1	RV Water Level	S/G #3 Level
Normal data	100						
Faulty data	92.98	60.04	68.65	83.05	93.20	93.47	87.55

**Table 3:** Comparative evaluation of imputation accuracy and computation time with symmetric mean absolute percentage error (sMAPE) metric.

Unit: %		Variable #1	Variable #2	Variable #3	Variable #4	Variable #5
Mean	Max/Min	79.98/8.82	198.00/108.34	198.81/3.13	82.04/4.75	63.04/2.77
	Average	39.94	195.35	135.97	18.06	30.09
KNN	Max/Min	34.35/0.01	82.73/0.00	76.96/0.00	56.00/0.00	21.15/0.00
	Average	3.93	1.94	3.25	4.21	2.50
MICE	Max/Min	22.65/2.79	198.87/11.18	198.41/3.26	6.34/0.0042	51.80/0.86
	Average	7.63	177.95	95.91	**0.21**	6.80
Missforest	Max/Min	14.93/0.076	44.61/0.00	38.27/0.00	23.67/0.0008	17.99/0.00
	Average	**1.68**	**1.41**	**0.27**	0.77	**1.48**

**Table 4:** Total average sMAPE and computation time of imputation methods.

	Mean	KNN	MICE	Missforest
**sMAPE**	83.88	3.17	57.70	1.12
**Computation time**	0.0198 s (SD = 2.45 × ^−4^)	17.31 s (SD = 1.24 × ^−1^)	4.53 s (SD = 1.53 × ^−1^)	9.06 s (SD = 2.33)

**Table 5:** Total diagnosis accuracies.

	Normal Data	Faulty Data	GRUD	Missforest
Total	100% (453/453)	82.71% (13,113/15,855)	96.75% (3068/3171)	100% (3171/3171)
